# IST-Yeasts CC: A Newly Established Culture Collection of Yeasts of Biotechnological Potential, Isolated from Algae Associated-Environments

**DOI:** 10.3390/bioengineering13070807

**Published:** 2026-07-14

**Authors:** Mónica A. Fernandes, Madalena Matos, Isabel Sá-Correia

**Affiliations:** 1 iBB-Institute for Bioengineering and Biosciences, Instituto Superior Técnico, Universidade de Lisboa, Av. Rovisco Pais, 1, 1049-001 Lisbon, Portugal; monica.a.fernandes@tecnico.ulisboa.pt (M.A.F.); madalena.matos@tecnico.ulisboa.pt (M.M.); 2 Associate Laboratory i4HB-Institute for Health and Bioeconomy at Instituto Superior Técnico, Universidade de Lisboa, Av. Rovisco Pais, 1, 1049-001 Lisbon, Portugal; 3 Department of Bioengineering, Instituto Superior Técnico, Universidade de Lisboa, Av. Rovisco Pais, 1, 1049-001 Lisbon, Portugal

**Keywords:** marine yeasts, blue biotechnology, biosurfactants, carotenoids, microbial oils, auxins, circular bioeconomy, yeast-algae co-cultivation

## Abstract

This article presents the IST-Yeasts Culture Collection (IST-Yeasts CC), which is a newly established repository dedicated to non-conventional blue yeasts isolated from (micro)algae-associated environments in Portugal. This collection currently comprises 115 yeast strains, the majority of which belong to the phylum Basidiomycota (92%), the genus *Rhodotorula* (69%), including *R. mucilaginosa*, *R. diobovata*, *R. sphaerocarpa*, and *R. taiwanensis* species. Other Basidiomycota species in the collection are: *Cystobasidium minutum*, *C. slooffiae*, *Vishniacozyma carnescens*, *Moesziomyces aphidis*, *Sporobolomyces roseus*, *S. salmonicolor*, and *Naganishia diffluens*. The collection also includes species from the Ascomycota phylum, such as *Meyerozyma guilliermondii*, *Yamadazyma atlantica*, and *Cyberlindnera vartiovaarae*. An initial functional screening performed, with one representative isolate per species, revealed the ability of these strains to produce carotenoids, lipids, riboflavin, biosurfactants, bioemulsifiers and auxins, as well as their capacity to grow on a broad range of carbon sources. These traits underscore their biotechnological potential within the circular bioeconomy and sustainable bioprocesses, positioning them as promising sources of bioactive natural products. By providing access to a diverse panel of marine-associated yeasts, the IST-Yeasts CC supports the development of innovative solutions in marine biotechnology and marine drug discovery, including the design of more resilient and high-performance algal cultivation systems through targeted co-cultivation strategies. Further information on the collection can be found at IST-Yeasts CC website.

## 1. Introduction

Yeasts are integral components of microbial ecosystems and have been increasingly recognized for their biotechnological potential [1,2,3,4,5]. Exploring their diversity across a wide range of environments is essential for identifying naturally robust strains capable of withstanding bioprocess stresses and exhibiting valuable catabolic and biosynthetic abilities [4,6,7,8]. Such isolates provide strong foundations for genetic enhancement, and their selection, together with optimized physiological and bioprocess conditions tailored to specific production goals, is crucial for maximizing productivity and ensuring economic viability [4,8,9,10,11,12,13]. As a result, strain-improvement strategies are most effective when applied to inherently resilient yeasts with advantageous metabolic profiles [12,14].

In recent years, interest in novel yeast isolates has intensified, particularly in the diverse and largely underexplored ecosystems associated with marine environments [3,6,15,16]. Marine habitats, especially those linked to algae, harbor extensive untapped microbial diversity and host microorganisms with unique metabolic pathways and notable resilience to harsh conditions such as high salinity and fluctuating temperatures [3,7,17,18]. Yeasts adapted to these environments often synthesize valuable metabolites, positioning them as promising candidates for biotechnological applications [7,16,19].

Marine algae, functioning as dynamic interfaces between the ocean and microbial communities, support interactions with yeasts with specialized biosynthetic and catabolic capabilities [7,16,20,21,22]. These microorganisms hold significant potential for the production of biofuels, pharmaceuticals, enzymes, and other high-value compounds, while leveraging sustainable marine resources [3,6,16,23,24]. Investigating yeast isolates from algal-associated environments therefore opens new avenues for environmentally friendly, sustainable bioprocessing and the development of novel industrial products [3,7,15,16,23,25,26]. In this context, studying marine algae-associated yeast microbiomes is both a scientific pursuit and a key step toward unlocking nature’s hidden biotechnological resources [9,11,12,13,16,27].

**Figure 1:**
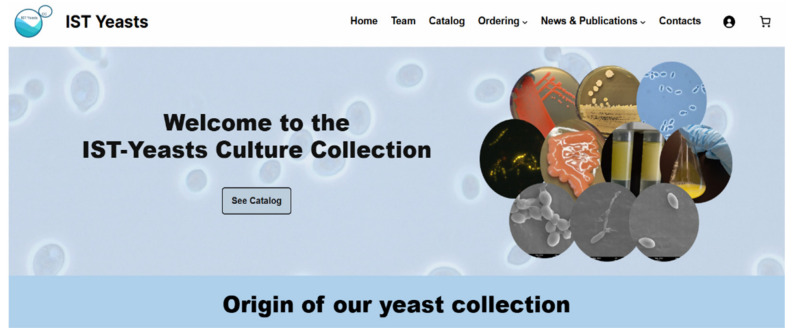
IST-Yeasts Culture Collection website homepage. https://blueyeastscc.tecnico.ulisboa.pt.

Despite their promise, marine- and algae-associated yeasts remain underrepresented in global culture collections. The IST-Yeasts Culture Collection (IST-Yeasts CC), based at the Biological Sciences Research Group of iBB—Institute for Bioengineering and Biosciences, Instituto Superior Técnico, University of Lisbon—is actively addressing this gap. Since its creation in 2024, in the framework of Blue Bioeconomy Pact project, 115 yeast strains were isolated, molecularly identified [5,28] and included in this culture collection (https://blueyeastscc.tecnico.ulisboa.pt) (Figure 1). On the culture collection website, the strain catalogue and services provided by IST-Yeasts CC can be consulted, as well as related news. Information on yeasts preserved in the IST-Yeasts CC is incorporated in the ARCTOS platform under the Portuguese Blue Biobank. ARCTOS is a collaborative, open-source collection management system that serves as a trusted provider of research-grade data for natural and cultural history collections [29].

This article aims to disseminate knowledge about the curated microbiological resource IST-Yeasts CC, providing prospective users with a comprehensive overview of its main features, the taxonomic diversity of the included yeast species, and their biotechnological potential. The IST-Yeasts CC comprises a diverse set of non-conventional yeast strains isolated from estuarine and marine environments, often in association with microalgae and macroalgae. To support this objective, a preliminary screening was conducted on one representative isolate of each species in the collection. This screening evaluated their capacity to produce lipids, auxins, carotenoids, vitamins and biosurfactants/bioemulsifiers, as well as their ability to assimilate relevant carbon sources. These traits form the foundation of their potential use as microbial cell factories, advancing a more sustainable circular bioeconomy.

The results highlight the potential of the yeast strains preserved and is a contribution to the Portuguese blue bioeconomy initiative. Continued exploration of these strains by the research community will deepen scientific understanding of marine-associated yeast diversity and unlock further biotechnological applications, as many species and their functional metabolites remain underexplored and show promising ecological and industrial potential.

## 2. Materials and Methods

### 2.1. Sampling Sites

For yeast isolation, culture samples were collected from industrial algal cultures from marine/estuarine environments in southern and central Portugal. Microalgal cultures of *Nannochloropsis oceanica*, *Microchloropsis gaditana* and *Tisochrysis lutea* produced at Necton S.A. (Olhão, Portugal), and the macroalgal surfaces of *Porphyra dioica*, industrially produced at ALGAplus (Aveiro, Portugal) were sampled and used for yeast isolation. Microalgal samples were collected before and after passage to new bioreactors during the industrial scale-up cultivation during two to four months. More details on *M. gaditana* and *T. lutea* samples can be found in [5,28]. Samples of *P. dioca* were collected from tanks containing five different life stages. Algal culture systems—*Limnospira maxima* and *Haematococcus* sp.—from the Algoteca of Faculty of Sciences, University of Lisbon were also sampled for yeast isolation.

### 2.2. Yeast Isolation and Preservation

Yeast isolation was achieved through an optimized protocol developed and described by [5,28]. Briefly, YPD medium plates [10 g/L yeast extract (VWR Chemicals, Radnor, PA, USA), 20 g/L peptone (BD Gibco, Waltham, MA, USA), 20 g/L glucose (Scharau, Barcelona, Spain), and 20 g/L agar (LabChem, Zelienople, PA, USA)], prepared with 50% (*v*/*v*) Artificial Sea Water [ASW; 23.38 g/L NaCl (PanReact, Barcelona, Spain), 2.41 g/L MgSO_4_·7H_2_O (LabChem), 1.9 g/L MgCl_2_·6H_2_O (Fluka Analytical, Buchs, Switzerland), 1.11 g/L CaCl_2_·2H_2_O (Merck, Darmstadt, Germany), 0.75 g/L KCl (Merck), 0.17 g/L NaHCO_3_ (LabChem) and ddH_2_O] and supplemented with 100 µg/mL chloramphenicol (Chl; Sigma, Burlington, MA, USA) were used to isolate yeasts. To attain higher yeast concentrations, the samples were processed in different ways. Samples (100 µL) from microalgal cultures were directly plated or plated after concentration by centrifugation (13,000× *g* for 10 min). Additionally, an enrichment culture was prepared by inoculation of the YPD liquid medium with the initial culture to a final concentration of 10% (*v*/*v*). The enrichment culture was incubated for two days at 22 °C and then plated as described before. Macroalgal samples were swabbed for retrieval of superficial yeasts or were sliced and mixed with macroalgal culture water and plated. After plate incubation at 22 °C for up to 10 days, several colonies of different morphology were retrieved and manipulated until pure cultures were obtained. Selected yeast isolates were cryopreserved in glycerol stocks [15% (*v*/*v*)] at −80 °C.

### 2.3. Molecular Identification at the Species Level

Molecular identification of yeast isolates was achieved as described before [5,28]. Briefly, genomic DNA was extracted following a method adapted from [30] to be used as template to amplify the D1/D2 domain sequence of the 28S ribosomal DNA (rDNA) and the internal transcribed spacer (ITS) region of rDNA. The primer pairs used for D1/D2 and ITS amplification were, respectively, NL-1 (5′-GCATATCAATAAGCGGAGGAAAAG-3′) and NL-4 (5′-GGTCCGTGTTTCAAGACGG-3′), and ITS1 (5′-TCCGTAGGTGAACCTGCGG-3′) and ITS4 (5′-TCCTCCGCTTATTGATATGC-3′), which are considered effective for the taxonomic identification of yeasts [31]. The yeast species were identified by running a nucleotide BLAST (http://www.ncbi.nlm.nih.gov/blast, assessed on 12 September 2024) with the D1/D2 and ITS-obtained amplified nucleotide sequences against the core nucleotide NCBI database. All D1/D2 and ITS sequences were submitted to GenBank (accession numbers available in https://blueyeastscc.tecnico.ulisboa.pt/catalogo/).

### 2.4. Phylogenetic Analysis

The phylogenetic placement of the 115 yeast isolates preserved in the IST-Yeasts CC was obtained through iterative alignment of the D1/D2 consensus rDNA sequences of the yeast isolated with the sequences of the type strains for each species (Supplementary Material Table S1). Multiple alignment was carried out with Muscle, available in the software MEGA-X v.11. The Mega-X software was also used for the phylogenetic tree construction using the maximum likelihood method of the Kimura 2-parameter model, selected based on the MEGA-X software recommendation considering the data [32,33]. The confidence level of the clades was estimated using a bootstrap analysis with 500 replicates.

### 2.5. Functional Characterization of Isolates

For phenotypic characterization, one isolate of each yeast species was selected at random (Table 1). Yeast cells were pre-cultured in liquid YPD medium for 24 h with orbital agitation (220 rpm), at 22 °C. Pre-cultured cells were harvested by centrifugation at 4600× *g* for 5 min at 4 °C and inoculated in 20 mL of a minimal medium [6.7 g/L of Yeast Nitrogen Base (BD Difco) and ddH_2_O with pH adjusted to 5.5] in 100 mL shake flasks, using an initial Optical Density at 600 nm (OD600nm) of 1. To test assimilation of different carbon sources the minimal medium was supplemented with different carbon sources: 20 g/L glucose or 20 g/L xylose (Sigma) or 20 g/L inulin (Sigma) or 1.5% (*v*/*v*) methanol (VWR) or 1.5% (*v*/*v*) glycerol (Sigma). These media were filter-sterilized using a 0.2 µm filter (Whatman^®^ Puradisc, Maidstone, UK). The cultures were incubated at 22 °C with orbital agitation (220 rpm), and growth was monitored by measuring culture OD600nm using a U-2000 HITACHI spectrophotometer (Tokyo, Japan). *Kluyveromyces marxianus* IST389 (formerly *K. fragilis* IGC 2671), a selected strain for the direct conversion of inulin to ethanol [34], and *Saccharomyces cerevisiae* BY4741, a well-known laboratory strain used as a parent strain for the international systematic *Saccharomyces cerevisiae* gene disruption project, were used as positive and negative controls, respectively, for inulin and xylose assimilation.

Carotenoid pigment and EPS production was evaluated visually in cultures grown on YPD medium and in colonies formed on YPD agar plates. Carotenoid pigment production was also observed in liquid media when the cultures reached the same Optical Density. Riboflavin production was previoulsy evaluated visually in liquid minimal media and confirmed following the method described in [35]. The following methods were used to assess the ability of yeast cells grown on the different carbon sources tested to produce lipids, biosurfactants/emulsifiers and auxins. Lipid accumulation was assessed with Nile Red staining method, a method that utilizes Nile Red fluorescence dye that binds with lipids in the cell (emission/excitation at 625/535 nm) [5]. Biosurfactant production was tested using the oil dispersion test and the emulsification index after 144 h of cultivation [5]. The oil dispersion tested was carried out by measuring the diameter of the halo formed after the addition supernatant of the culture on top of a car oil mixture. The emulsification index was performed measuring the emulsification layer formed after vigorous mixing of the supernatant with olive oil. Auxin production was assessed using the Salkowski Reagent method, a colorimetric method that allows measurement of the indole-3-acetic acid (IAA) concentration [28].

## 3. Results

### 3.1. Yeast Composition of the IST-Yeasts Culture Collection

**Figure 2:**
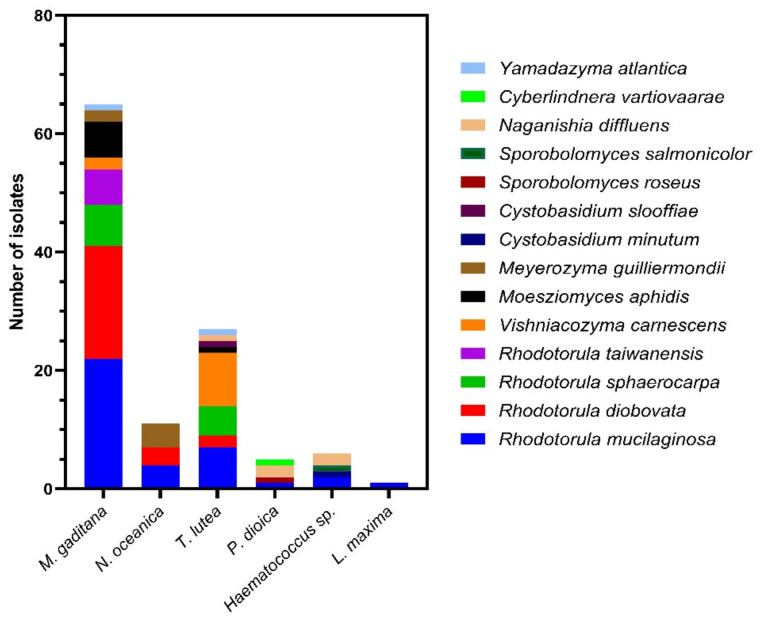
Number of the yeast isolates of different species preserved in the IST-Yeasts Culture Collection that were obtained from different algal origins, specifically, from the microalgae *Microchloropsis gaditana*, *Tisochrysis lutea* and *Nannochloropsis oceanica*, cultivated at Necton S.A facilities (Olhão, Portugal), the macroalgae *Porphyra dioica* cultivated at ALGAplus (Aveiro, Portugal), and the *Limnospira maxima* and *Haematococcus* sp. cultures grown at the Algoteca of Faculty of Sciences, University of Lisbon.

The distribution of yeast isolates preserved in the IST-Yeasts CC, among different species and their algal origin, is shown in Figure 2.

**Figure 3:**
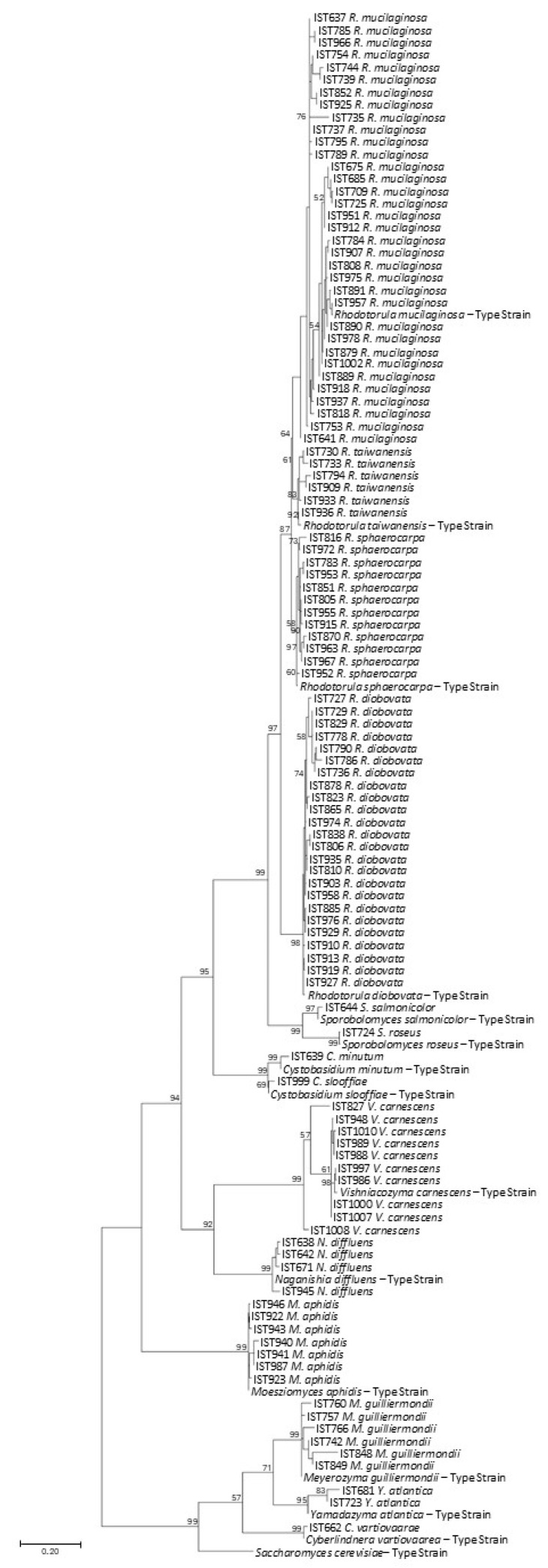
Phylogenetic analysis of yeast isolates included in the IST-Yeasts CC. The phylogenetic analysis was based on the alignment of sequences of the D1/D2 domain of the 28S rDNA region (accession numbers to Genbank in the IST-Yeasts CC catalogue), inferred by means of the maximum likelihood method and Kimura 2-parameter model. Sequences from the type strains of the different yeast species were included. The scale bar indicates the number of expected substitutions per site. The numbers provided at the branches are the frequencies (in percentage) of appearance of a given branch in 500 bootstrap replications.

A total of 115 yeast isolates, belonging to 14 species, are preserved in the IST-Yeasts CC. Of these, 92% belong to the Basidiomycota phylum. The genus *Rhodotorula* (*R. mucilaginosa*, *R. diobovata*, *R. sphaerocarpa*, and *R. taiwanensis*) is the most represented genus, comprising 69% of the isolates. Other Basidiomycota species in the collection include *Cystobasidium minutum*, *C. slooffiae*, *Vishniacozyma carnescens*, *Moesziomyces aphidis*, *Sporobolomyces roseus*, *S. salmonicolor*, and *Naganishia diffluens*. The species from the Ascomycota phylum included in the culture collection are *Meyerozyma guilliermondii*, *Yamadazyma atlantica*, and *Cyberlindnera vartiovaarae*. The species *Y. atlantica* and *C. vartiovaarae* were formerly classified within the *Candida* genus. A detailed on-line catalog (https://blueyeastscc.tecnico.ulisboa.pt/catalogo/) with information on the yeast isolates is available at the IST-Yeasts CC website. The results of the phylogenetic analysis of these yeast isolates are presented in Figure 3. Although the isolates included in the IST-Yeasts CC do not fully represent the yeast populations of each sampling site, they were selected to preserve the diversity and relative abundance of species recorded at those locations. A full description of the culturable yeasts recovered from *M. gaditana* and *T. lutea* can be found in [5,28], respectively. In addition to dominating the overall culture collection, *Rhodotorula* species were prevalent across all algal cultures analyzed (Figure 2). Notably, *Rhodotorula mucilaginosa* was isolated from every sampled algae-associated environment. Although less abundant, several other species were detected in association with different algal hosts. For example, *Naganishia diffluens* was identified in samples from Necton S.A., ALGAplus, and Algoteca, encompassing diverse algal species and geographical locations.

### 3.2. Functional Properties

The 14 yeast species included in the IST-Yeasts CC have potential for a wide range of biotechnological applications. Based on the information available in the literature and on our previous works [5,28], selected functional properties of each yeast species were assessed. This work was required since several of the non-conventional yeast species included in IST-Yeasts CC are understudied and there is no relevant information in the literature about their application potential. One isolate per species was selected, at random. This approach was adopted to provide a broad overview of potential traits present across the taxonomic diversity of the collection, while keeping the screening effort manageable given the large number of strains.

#### 3.2.1. Assimilation of Relevant Carbon Sources

To assess the capacity of isolates from different species to utilize carbon sources relevant to a circular bioeconomy, one representative of each species (Table 1) was cultured for 72 h at 22 °C in minimal medium with a single C-source: glucose (20 g/L), xylose (20 g/L), inulin (20 g/L), methanol [1.5% (*v*/*v*)] or glycerol [1.5% (*v*/*v*)].

**Figure 4:**
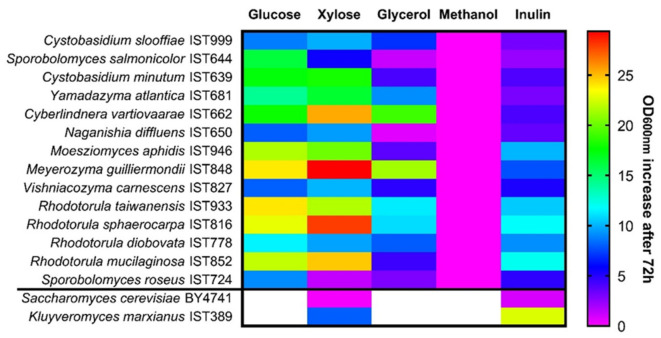
Carbon source assimilation by IST-Yeasts CC strains of different species. The data shown represents the increase in the culture OD600nm from 0 h to 72 h of cultivation in minimal medium supplemented with the 20 g/L glucose, 20 g/L xylose, 20 g/L inulin, 1.5% (*v*/*v*) methanol or 1.5% (*v*/*v*) glycerol, as described in Section 2. *Kluyveromyces marxianus* IST389, *Meyerozyma guilliermondii* IST848 and *Saccharomyces cerevisiae* BY4741 as controls for growth in mediawith inulin, glycerol and xylose assimilation.

The species present in the IST-Yeasts CC were, in general, capable of robust growth on glucose or xylose, although reaching different final biomass concentrations after 72 h of cultivation (Figure 4). The hydrolysis of lignocellulosic feedstock releases fermentable sugars such as glucose and xylose, which can be utilized for bioproduct production. For example, metabolically suitable strains capable of efficiently assimilating both sugars can convert them into oils and other value-added compounds [4]. The fermenting yeast *S. cerevisiae*, used as a negative control, cannot naturally assimilate xylose, which is a limitation to the production of bioethanol and other bioproducts from lignocellulosic hydrolysates [4]. Except for *Sporobolomyces roseus*, all the isolates in the IST-Yeasts CC were able to grow on xylose as the sole carbon source. Several species attained a higher final biomass concentration with xylose compared with glucose, among them, *M. guilliermondii* and *R. sphaerocarpa.* None of the isolates were native methylotrophs, capable of assimilating methanol, an emerging, low-cost, and non-food single-carbon feedstock in industrial biotechnology, representing a highly reduced and abundant substrate to produce biofuels and high-value chemicals [36].

Cultivation on inulin as a sole carbon source led to low final biomass concentrations for several species, although slightly above the value corresponding to the negative control *Saccharomyces cerevisiae* BY4741, in particular for the species *Cystobasidium slooffiae*, *Sporobolomyces salmonicolor*, *C. minutum*, *Yamadazyma atlantica*, *Cyberlindnera vartiovaarae* and *Naganishia diffluens*. Strains of the *Rhodotorula* species and *M*. *aphidis* exhibited significant growth on inulin, although the final biomass concentration attained was below the one registered for the positive control, *Kluyveromyces marxianus* IST389. This strain, formerly *K. fragilis* IGC 2671, was selected for highly efficient direct production of ethanol from inulin, a fructan polymer present in Jerusalem artichoke juice [34]. Jerusalem artichoke is characterized by high tuber productivity and its use in consolidated bioprocessing with *Kluyveromyces marxianus* for ethanol fermentation shows strong potential as a bioenergy feedstock, achieving ethanol yields comparable to those obtained from corn and sugarcane [37]. Glycerol assimilation was also evaluated. *Cyberlindnera vartiovaarae* and *Meyerozyma guilliermondii* achieved the highest final biomass concentrations on this carbon source, followed by *Rhodotorula taiwanensis* and *R. sphaerocarpa*. In contrast, *Sporobolomyces salmonicolor* and *Naganishia diffluens* showed no detectable growth on glycerol. Since *Meyerozyma guilliermondii* is known to efficiently assimilate glycerol [38], this isolate was used as a positive control.

#### 3.2.2. Potential to Produce Value-Added Bioproducts: Lipids, Biosurfactants/Bioemulsifiers, Carotenoids and Auxins

**Figure 5:**
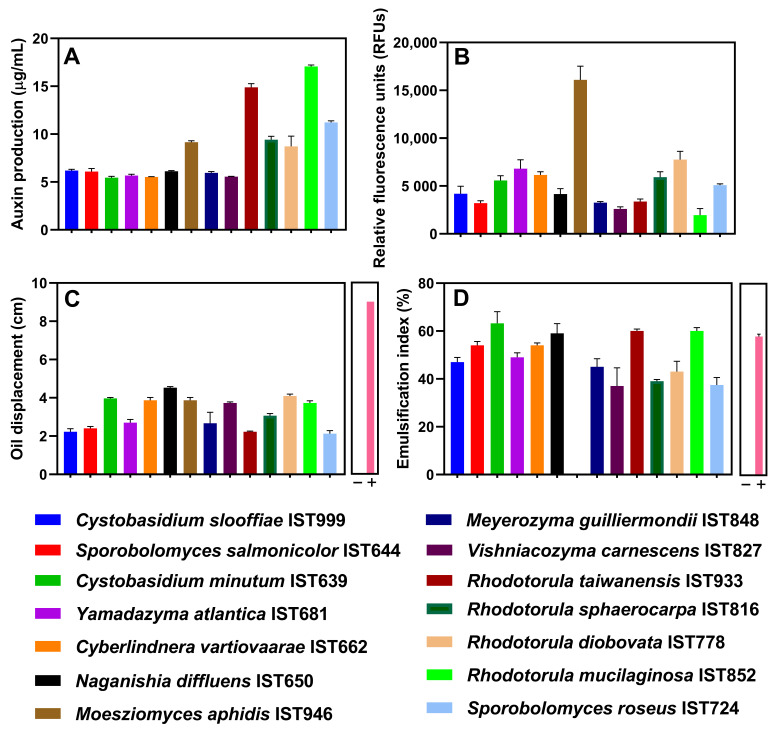
Auxin, lipid, and biosurfactant and bioemulsifier production by a selected strain of each yeast species present in the IST-Yeasts CC when glucose (20 g/L in minimal medium) was used as carbon source. Auxin production (**A**) was assessed after 72 h of cultivation using the Salkowski Reagent method. Lipid production (**B**) was assessed by Nile Red staining after 48 h and is indicated in relative fluorescence units (RFUs). Biosurfactant and bioemulsifier production was assessed through the oil displacement method (**C**) and the emulsification index (**D**), respectively, after 144 h. For the biosurfactant and bioemulsifier assessment, the positive control was a solution of 1% SDS (*w*/*v*) and the negative control was the sterile growth medium. Data represents the average of three independent experiments, and the error bars indicate standard deviation.

**Figure 6:**
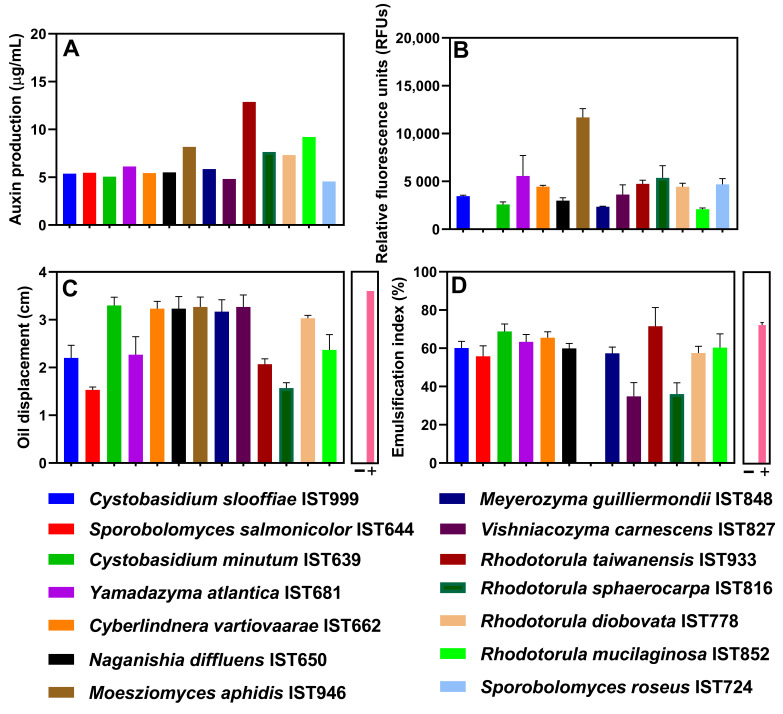
As for Figure 5, except for the carbon source used for cultivation, which was xylose (20 g/L) instead of glucose.

**Figure 7:**
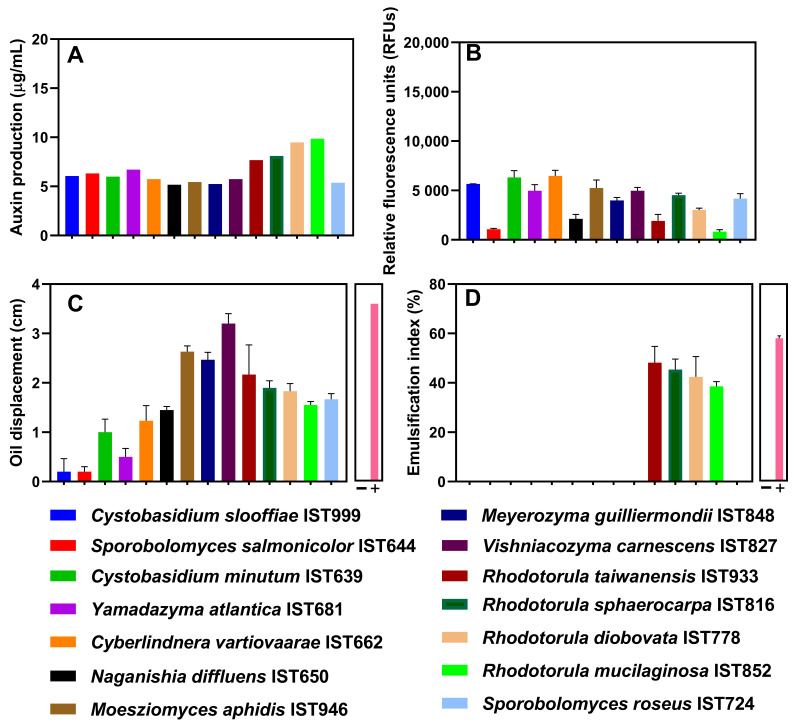
As for Figure 5, except for the carbon source used for cultivation, which was inulin (20 g/L) instead of glucose.

**Figure 8:**
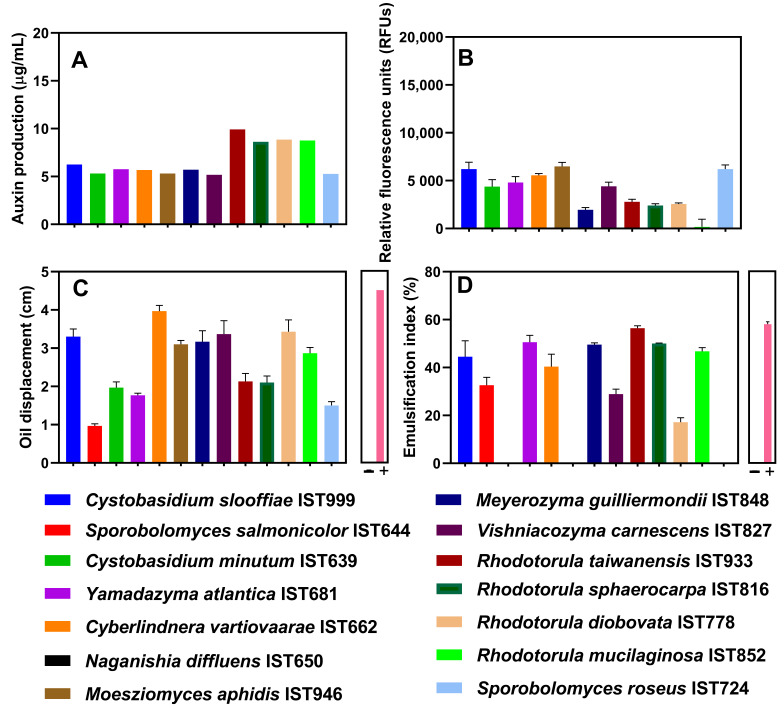
As for Figure 5, except for the carbon source used for cultivation, which was glycerol [1.5% (*v*/*v*)] instead of glucose.

The same selected isolates of each species (Table 1) were used for assessment of their capacity to produce lipids, biosurfactants/bioemulsifiers and auxins in minimal medium with glucose as a carbon source (Figure 5). This biotechnological potential was also assessed when alternative carbon sources were used (Figure 6, Figure 7 and Figure 8). Selected isolates were cultured in shake flasks at 22 °C under standardized conditions in a defined medium supplemented with each carbon source, adjusted to pH 5.5, as described in Section 2 In some cases, no production was recorded despite significant growth (for example, *M. aphidis* bioemulsification activity). In a few cases the lack of production was due to marginal growth after the standardized cultivation time, for example, *Sporobolomyces salmonicolor*’s poor growth on glycerol after 48 and 72 h implicated no lipid or auxin detectable production.

**Figure 9:**
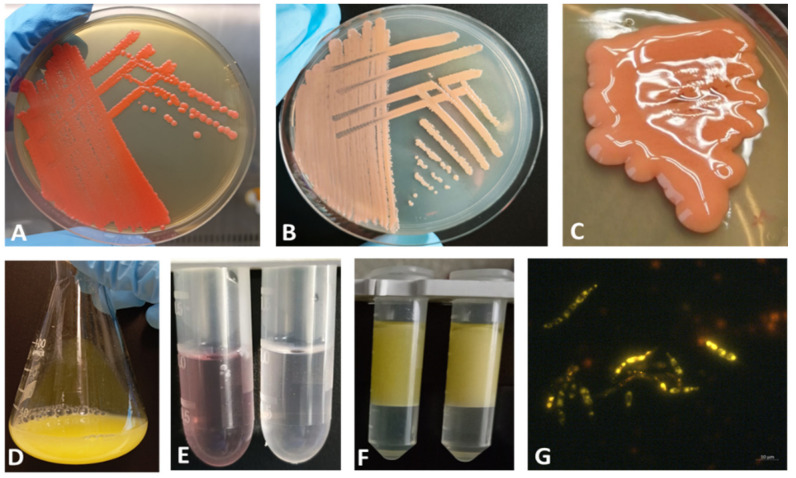
Examples of demonstration of biotechnologically relevant properties. YPD-agar-plate culture of (**A**)—*Rhodotorula taiwanensis*; (**B**)—*R. diobovata*; and (**C**)—*R. mucilaginosa*. (**D**)—Liquid culture of *Meyerozyma guilliermondii* showing a yellow color due to riboflavin production. (**E**)—Analysis of auxin production by *R. sphaerocarpa* (**left**), assessed by the Salkowski Reagent method. (**F**)—Production of emulsifier by *R. mucilaginosa* (**left**) and *R. diobovata* (**right**). (**G**)—Cells of *Moesziomyces aphidis* with lipid droplets stained with Nile Red, observed in a fluorescence microscope.

Carotenoid production was assessed by visual observation of culture growth on YPD agar plates, incubated under the same conditions. Carotenoid production was also verified in liquid media when the culture reached the same Optical Density. As expected, *Rhodotorula* species, as well as *Sporobolomyces* and *Cystobasidium* species, were able to produce carotenoids given the pink-red coloration observed. It is known that torularhodin, γ-carotene, torulene and β-carotene are the main types of carotenoids produced by those species, being produced in different proportions according to the species [39,40,41,42]. Differences observed in the coloration across different species (e.g., Figure 9A,B) and even across different isolates of the same species (e.g., isolates of *Rhodotorula mucilaginosa*), suggest different types/proportions of carotenoids being produced [39,40,41,42].

Several strains of the IST-Yeasts CC, especially *R. mucilaginosa* strains (Figure 9C), are known to produce exopolysaccharides (EPSs) [43,44]. This is consistent with the mucoid texture of *R. mucilaginosa* strain colonies on YPD-agar plates. However, EPS production was not systematically assessed in our study.

*Meyerozyma guilliermondii* is a known riboflavin producer [45]. In fact, when cultured in liquid minimal medium, the culture develops a yellow coloration typical of riboflavin (Figure 9D). Riboflavin production was previoulsy confirmed by a colorimetric method, mixing the supernatant with HCl and measuring absorbance at 445 nm.

Auxin (indole-3-acetic acid) production was assessed with the Salkowski Reagent method (Figure 9E). In general, the results are similar across the different carbon sources tested (Figure 5A, Figure 6A, Figure 7A and Figure 8A). Nevertheless, in medium with glucose, only *R. taiwanensis* and *R. mucilaginosa*’s isolates attained an auxin concentration close to 15 µg/mL. A recently published study by our team [28] tested auxin production in a culture medium with glucose as a carbon source for several yeast isolates obtained from an industrial culture of the microalgae *Tisochrysis lutea*. Although the production level was strain-dependent, with isolates of the same species being better producers than others, *Rhodotorula mucilaginosa* and *R. sphaerocarpa* isolates were also found to produce the most significant amounts of auxin [28].

Lipid production (Figure 5B) was assessed with the fluorescence dye Nile Red. When cultured in a medium with glucose or xylose, the *Moesziomyces aphidis* isolate exhibited the higher fluorescence values consistent with conclusions from previous studies [5]. Microscopic observation confirmed the presence of lipid bodies in *M. aphidis* cells (Figure 9G). *C. vartiovaarae* and *M. guilliermondii* isolates achieved a high final biomass with glycerol as a carbon source, however this did not result in higher lipid production when compared to growth on glucose. Interestingly, *Cystobasidium* isolates, when using inulin as a carbon source, could not achieve a biomass as high as with glucose but lipid production levels were higher (Figure 6B).

Biosurfactant production was assessed based on the oil displacement test (Figure 5C, Figure 6C, Figure 7C and Figure 8C). In general, under the tested conditions all isolates tested were able to produce biosurfactants. Although exhibiting weak oil displacement activity compared with the solution used as a positive control, the levels are significant. Bioemulsifier production was assessed based on the emulsification index (Figure 5D, Figure 6D, Figure 7D and Figure 8D). The *Rhodotorula mucilaginosa*, *R. diobovata*, *Meyerozyma guilliermondii* and some *R. taiwanensis* isolates tested produced biosurfactants with emulsifying properties, as also reported before [5]. In the present study, all species were found to be able to produce bioemulsifiers on glucose (Figure 5D), except for *Moesziomyces aphidis*. When using a medium with inulin, the bioemulsifier production was only observed for the *Rhodotorula* species (Figure 6D). *Rhodotorula* species have been reported to produce biosurfactants/bioemulsifiers from the glycolipids class, more particularly sophorolipids [46]. *M. guilliermondii* has also been described as a producer of biosurfactants with emulsifying properties, also from the glycolipid class [47]. *M. aphidis* has been extensively described as producing a highly promising mannosylerythritol (MEL) biosurfactant [48]. However, no bioemulsifer activity was detected in this study, as reported previously using similar conditions [5]. Since MEL production was reported to be limited when monomeric sugars are used as the sole carbon source, usually requiring the addition of lipids to the growth media for high production, it is likely that the tested conditions were not the best choice [49].

## 4. Discussion

The IST-Yeasts Culture Collection presented here addresses an often overlooked yet influential component of marine microalgal systems: yeasts. By focusing on the preserved marine-associated strains, this collection may contribute to the future development of more sustainable bioprocesses within a circular bioeconomy framework. In contrast to the predominantly terrestrial bias of most existing yeast collections, the IST-Yeasts CC comprises 115 molecularly identified blue yeast strains. These strains exhibit the capacity to produce a broad spectrum of compounds with biotechnological relevance, as reported in the literature for several of the preserved species and, in several cases, confirmed, or observed for the first time, in the present study (Table 2).

The taxonomic composition and functional traits of the IST-Yeasts CC highlight the largely untapped potential of marine (micro)algae-associated yeasts as sources of bioactive metabolites and chemical mediators. The capacity of these yeasts to produce lipids (most species are oleaginous, with applications in biofuels, oleochemicals, and cosmetics) together with carotenoids, vitamins, biosurfactants, emulsifiers, and enzymes, emphasizes their relevance as microbial cell factories. In particular, pigmented Basidiomycota, dominated by isolates of the genus *Rhodotorula*, combine the production of these high-value metabolites with rapid growth on diverse carbon sources, including abundant low-cost substrates and residues [4,50,51,52]. The collection also includes several isolates of *Moesziomyces aphidis*, a well-known producer of mannosylerythritol lipids (MELs). These biosurfactants combine low critical micelle concentrations with high environmental compatibility, supporting their use in sustainable and circular bioeconomy processes [48,49]. The assessment of the capacity of the *M. aphidis* isolate to produce biosurfactant was likely underestimated in this work. This species requires a hydrophobic substrate (e.g., soybean oil) in the growth medium to trigger high yields and extracellular excretion of surface-active MELs [49]. In our study, a water-soluble substrate (glucose, xylose, glycerol and inulin) was used without an oil and the pathway for biosurfactant production and excretion may not have been fully induced [49]. Remarkably, no bioemulsifying activity was detected in *M. aphidis* cultures while biosurfactant activity was, as described before [5]. While both are surface-active, amphiphilic molecules produced by microbes, their mechanisms and physical properties differ significantly and a biosurfactant is not necessarily a bioemulsifier [53].

Beyond their biosynthetic capacity for added-value compounds, several isolates in the collection produce auxins (e.g., *Rhodotorula mucilaginosa* and *R. sphaerocarpa* isolates), positioning these molecules as key chemical signals in marine microalgae–yeast interactions [16,28,54]. From a chemical-ecology perspective, auxins may act as interkingdom signaling molecules that modulate algal growth, cellular differentiation, and stress responses, thereby shaping community structure and functional performance [54,55]. The recurrent detection of auxin-producing yeasts within (micro)algae-associated environments suggests that these phytohormones constitute an adaptive chemical strategy, mediating mutualistic or facilitative interactions [16,28]. Harnessing such signaling functions through controlled co-cultivation may enable the rational design of more resilient and productive algal–yeast systems, with implications for metabolite induction and bioprocess optimization [56,57].

As reported before, the ability of isolates of *Rhodotorula sphaerocarpa*, *R. mucilaginosa*, *R. diobovata*, *Naganishia diffluens*, and *Moesziomyces aphidis* to grow optimally at moderate temperatures (25–30 °C), together with the psychrotolerant profile of *Vishniacozyma carnescens* [28], highlights the adaptability of isolates gathered in the Yeast Collection to diverse environmental and process conditions. Moreover, also as reported before [28], the broad tolerance of isolates of those species to artificial seawater, combined with the observed stimulation of growth in *Moesziomyces guilliermondii* and *Rhodotorula sphaerocarpa*, underscores their suitability for cultivation in saline- or seawater-based media. These traits support their potential for biotechnological applications requiring robust performance under saline conditions, including marine-based bioprocesses and sustainable production systems that reduce reliance on freshwater resources.

The assessment of only one representative isolate per species does not allow general conclusions about the well-documented phenomenon of intraspecific variability or the full biotechnological potential of the species or the strain collection. However, the present study provides a preliminary overview of the collection, illustrating the potential of some selected traits of representative isolates. We expect that researchers interested in the specific functional properties will use these resource to explore the full diversity within species and perform more detailed analyses on multiple strains, thereby capturing the variability and maximizing the discovery of biotechnologically relevant traits positioning the IST-Yeasts CC as a valuable platform to support research and innovation through the investigation of marine yeast-derived bioactive compounds and signaling molecules at the interface of chemical ecology, biotechnology, and marine drug discovery.

**Table 2:** Biotechnological potential of the yeast species gathered in the IST-Yeasts CC, as reported in the literature and/or as assessed in our study.

Yeast Species	Biotechnological Potential	References
*Rhodotorula mucilaginosa*	Production of lipids, carotenoids, exopolysaccharides, enzymes, biosurfactants/bioemulsifiers, and auxins. Capable of using glucose, acetic acid, galactose, xylose, pectin, inulin, sucrose, lactose and glycerol as C-source.	[4,5,28,50,52,58,59,60,61] This work.
*Rhodotorula diobovata*	Production of lipids, carotenoids, biosurfactants/bioemulsifiers and auxins; nitrogen fixation. Capable of using glucose, xylose, inulin and glycerol as C-source.	[5,50,62,63] This work.
*Rhodotorula taiwanensis*	Production of lipids, carotenoids, biosurfactants/bioemulsifiers, and auxins. Resistant to acids, heavy metals and gamma radiation (bioremediation of acidic radioactive sites). Capable of using glucose, xylose, glycerol and inulin as C-source.	[4,5,27,50,64] This work.
*Rhodotorula sphaerocarpa*	Production of lipids, carotenoids, and auxins. Capable of using glucose, xylose, glycerol and inulin as C-source.	[5,28,50] This work.
*Moesziomyces aphidis*	Production of lipids and biosurfactants. Capable of using glucose, xylose, inulin, xylan, sucrose, fructose, xylose, arabinose and cellobiose as C-source.	[5,49,65,66] This work.
*Meyerozyma guilliermondii*	Production of lipids, riboflavin (vitamin B2), and enzymes. Copper tolerance and removal. Capable of using glucose, xylose, glycerol, palm acid oil, sucrose and fructose as C-source.	[5,18,22,45,67,68,69] This work.
*Vishniacozyma carnescens*	A poorly studied psychrotolerant yeast. Production of biosurfactants/bioemulsifiers. Capable of using glucose, xylose, lactose, erythritol and cadaverine as C-source.	[70,71,72] This work.
*Cystobasidium minutum*	Poorly studied yeast. Production of lipids and carotenoids, antibacterials, and biosurfactants/bioemulsifiers. Capable of using glucose, xylose, sucrose, fructose, lactose and maltose as C-source.	[73,74,75]. This work.
*Cystobasidium* *slooffiae*	A poorly studied yeast. Production of carotenoids and biosurfactants/bioemulsifiers. Capable of using glucose and xylose as C-source.	This work.
*Sporobolomyces roseus*	A poorly studied psychrotrophic yeast. Production of lipids, carotenoids, auxins, and exopolysaccharides. Capable of using glucose, sucrose and inulin as C-source.	[76,77,78,79] This work.
*Sporobolomyces salmonicolor*	Production of lipids, carotenoids, enzymes (e.g., lipases, reductases, proteases, pectinases and chitinases), exopolysaccharides, biosurfactants/bioemulsifiers and flavoring molecules (γ-Decalactone). Capable of using glucose and xylose as C-source.	[41,73,80,81,82,83] This work.
*Naganishia diffluens*	Poorly studied yeast. Production of lipids, biosurfactants/bioemulsifiers, and enzymes. Capable of using glucose, glycerol and xylose as C-source.	[84,85] This work.
*Cyberlindnera vartiovaarae*	Poorly studied yeast. Production of biosurfactants/bioemulsifiers. Capable of using glucose, glycerol and xylose as C-source.	This work.
*Yamadazyma atlantica*	Poorly studied psychrotrophic yeast. Production of biosurfactants/bioemulsifiers. Capable of using glucose, glycerol and xylose as C-source.	[24] This work.

## 5. Conclusions

The IST-Yeasts Culture Collection is a publicly accessible repository of non-conventional yeasts isolated from (micro)algae-associated environments in Portugal. It currently comprises more than 115 strains of 14 different species that have been identified using molecular techniques and initially characterized for selected functional and biotechnological traits. Dedicated to the preservation and distribution of marine yeast biodiversity, the collection supports research and innovation by enabling the exploration of marine-derived bioactive metabolites and promoting the development of sustainable biotechnological solutions. Continued exploration of these strains by the research community is expected to deepen our understanding of marine-associated yeast diversity and to further expand their biotechnological applications.

## Figures and Tables

**Table 1:** Selected isolates from IST-Yeasts CC used for biotechnological potential assessment with the corresponding accession numbers of the D1/D2 and ITS.

ID	Species	Isolation Sample	DI/D2 Accession Number (NCBI)	ITS Accession Number (NCBI)
IST946	*Moesziomyces aphidis*	*Microchloropsis gaditana* culture	PQ346816	PQ346858
IST848	*Meyerozyma guilliermondii*	*Microchloropsis gaditana* culture	PQ341232	PQ351482
IST827	*Vishniacozyma carnescens*	*Microchloropsis gaditana* culture	PP944311	PP952021
IST852	*Rhodotorula mucilaginosa*	*Microchloropsis gaditana* culture	PQ341236	PQ351486
ITS778	*Rhodotorula diobovata*	*Nannochloropsis oceanica* culture	PQ380553	PQ396209
IST933	*Rhodotorula taiwanensis*	*Microchloropsis gaditana* culture	PQ344309	PQ351587
IST816	*Rhodotorula sphaerocarpa*	*Microchloropsis gaditana* culture	PQ341202	PQ351452
IST650	*Naganishia diffluens*	*Porphyra dioica* culture	PP156551	PP158638
IST662	*Cyberlindnera vartiovaarae*	*Porphyra dioica* culture	PP156560	PP158647
IST681	*Yamadazyma atlantica*	*Tisochrysis lutea* culture	PP341345	PP341893
IST639	*Cystobasidium minutum*	*Haematococcus* sp. culture	PP116149	PP115447
IST644	*Sporobolomyces salmonicolor*	*Haematococcus* sp. culture	PP116153	PP115452
IST724	*Sporobolomyces roseus*	*Porphyra dioica* culture	PP156566	PP158653
IST999	*Cystobasidium slooffiae*	*Tisochrysis lutea* culture	PQ346855	PQ346897

## Data Availability

Information on all the strains and molecular sequence data (D1/D2 and ITS), submitted to GenBank, are available through the online catalog: (https://blueyeastscc.tecnico.ulisboa.pt/catalogo/). Strain access is granted under standard Material Transfer Agreements (MTAs).

## Supplementary Materials

The following supporting information can be downloaded at: https://www.mdpi.com/article/10.3390/bioengineering13070807/s1, Table S1: GenBank NCBI accession number of the D1/D2 of the type strains of the different species in IST-Yeasts CC.
