# Supplementary material for: IST-Yeasts CC: A Newly Established Culture Collection of Yeasts of Biotechnological Potential, Isolated from Algae Associated-Environments
Source: Bioengineering (Basel). 2026 Jul 14;13(7):807. doi: 10.3390/bioengineering13070807 (PMC13404600; doi:10.3390/bioengineering13070807)
Supplement: Supplementary file 1 [file bioengineering-13-00807-s001.zip › bioengineering-4405132-supplementary.pdf]

Table S1. GenBank NCBI accession number of the D1/D2 of the type strains of the different species in IST-Yeasts CC.

Species	NCBI Accession Number
Moesziomyces aphidis	NG_069796.1
Meyerozyma guilliermondii	NG_042640.1
Vishniacozyma carnescens	NG_058430.1
Rhodotorula mucilaginosa	NG_055716.1
Rhodotorula diobovata	NG_042340.1
Rhodotorula taiwanensis	NG_063944.1
Rhodotorula sphaerocarpa	NG_042342.1
Naganishia diffluens	NG_058351.1
Cyberlindnera vartiovaarae	NG_060381.1
Yamadazyma atlantica	NG_054857.1
Cystobasidium minutum	NG_059005.1
Sporobolomyces salmonicolor	NG_056268.1
Sporobolomyces roseus	NG_069417.1
Cystobasidium slooffiae	NG_059008.1
Saccharomyces cerevisiae	NG_042623.1
